# Identification of the Enterotoxigenic Potential of *Staphylococcus* spp. from Raw Milk and Raw Milk Cheeses

**DOI:** 10.3390/toxins16010017

**Published:** 2023-12-28

**Authors:** Patryk Wiśniewski, Joanna Gajewska, Anna Zadernowska, Wioleta Chajęcka-Wierzchowska

**Affiliations:** Department of Food Microbiology, Meat Technology and Chemistry, Faculty of Food Science, University of Warmia and Mazury, Plac Cieszyński 1, 10-726 Olsztyn, Poland; joanna.gajewska@uwm.edu.pl (J.G.); anna.zadernowska@uwm.edu.pl (A.Z.); wioleta.chajecka@uwm.edu.pl (W.C.-W.)

**Keywords:** staphylococci, *Staphylococcus aureus*, coagulase-negative staphylococci, staphylococcal enterotoxins, raw milk, raw milk cheeses, SET-RPLA

## Abstract

This study aimed to genotypic and phenotypic analyses of the enterotoxigenic potential of *Staphylococcus* spp. isolated from raw milk and raw milk cheeses. The presence of genes encoding staphylococcal enterotoxins (SEs), including the classical enterotoxins (*sea-see*), non-classical enterotoxins (*seg-seu*), exfoliative toxins (*eta-etd*) and toxic shock syndrome toxin-1 (*tst-1*) were investigated. Isolates positive for classical enterotoxin genes were then tested by SET-RPLA methods for toxin expression. Out of 75 *Staphylococcus* spp. (19 *Staphylococcus aureus* and 56 CoNS) isolates from raw milk (49/65.3%) and raw milk cheese samples (26/34.7%), the presence of enterotoxin genes was confirmed in 73 (97.3%) of them. Only one isolate from cheese sample (1.3%) was able to produce enterotoxin (SED). The presence of up to eight different genes encoding enterotoxins was determined simultaneously in the staphylococcal genome. The most common toxin gene combination was *sek*, *eta* present in fourteen isolates (18.7%). The *tst-1* gene was present in each of the analyzed isolates from cheese samples (26/34.7%). Non-classical enterotoxins were much more frequently identified in the genome of staphylococcal isolates than classical SEs. The current research also showed that genes tagged in *S. aureus* were also identified in CoNS, and the total number of different genes detected in CoNS was seven times higher than in *S. aureus*. The obtained results indicate that, in many cases, the presence of a gene in *Staphylococcus* spp. is not synonymous with the ability of enterotoxins production. The differences in the number of isolates with genes encoding SEs and enterotoxin production may be mainly due to the limit of detection of the toxin production method used. This indicates the need to use high specificity and sensitivity methods for detecting enterotoxin in future studies.

## 1. Introduction

According to the current European Commission Regulations (EC) No. 2073/2005, as amended, on microbiological criteria for foodstuffs [1], the hygiene criteria focus on examining of the number of staphylococci. The acceptable limit for these bacteria in food has been set at 10^5^ cfu/g, but it specifically addresses coagulase-positive staphylococci (CPS), excluding coagulase-negative staphylococci (CoNS), which are prevalent in many food products [1]. This fact affects the possibility that CoNS may have a significant role in the pathogenesis of food-borne diseases [2]. An annual report published by the European Food Safety Authority (EFSA) indicates a problem with CPS and enterotoxins (in milk and cheese, among others), with no data on CoNS [3].

Staphylococcal enterotoxins (SEs) are an extensive group of toxins included in the family of staphylococcal and streptococcal extrinsic pyrogenic toxins (PTs), sharing phylogenetic, structural, functional, or common sequence homology. These toxins are responsible for causing toxic shock syndrome, food poisoning, as well as some allergic reactions and autoimmune diseases [4]. Among them are toxic shock toxin 1 (TSST-1), exfoliative toxins (ETA–ETD), staphylococcal enterotoxins with emetic activity (SEA-SEE, SEG-SEI, SEK-SET, SEY) [5,6], and SE-like toxins (SElV, SElX, SElW, SElZ, SEl26, SEl27) [7,8,9]. Each of the enterotoxins has superantigenic activity, so they have been designated as staphylococcal superantigens (SAgs) [5,10,11]. SEs are soluble in water and salt solutions. Once supplied with food, they remain active in the digestive tract—they show high stability and resistance to most proteolytic enzymes, including trypsin and pepsin. They also show insensitivity to enzymes used in cheese production—renin, chymotrypsin or papain. They are characterized by high heat resistance and are resistant to unfavorable conditions for the growth of microorganisms (including heat treatment, low pH) [12]. For many years, a coagulase-positive *S*. *aureus* group representative was considered the only species capable of producing enterotoxins. However, scientists are increasingly focusing their research on the pathogenic potential of coagulase-negative staphylococci. Reports in recent years have indicated the enterotoxigenic potential of the CoNS group, which was isolated from raw milk samples, among others [2,13,14,15].

Staphylococcal enterotoxins show a range of negative activities. One of these includes emesis activity. An appropriate concentration of a given enterotoxin in the ingested food is required to produce the adverse effect. The exact values have not yet been investigated due to the lack of a suitable animal model reflecting the course of poisoning occurring in humans [16]. Detection of enterotoxins in food is crucial to maintain appropriate levels of food safety. Methods based on polyvalent enzyme-linked immunoassays (ELISA) or enzyme-linked fluorescent assay (EFLA) with antibodies that detect SEA-SEE have been used to detect SEs in food [17]. These methods do not detect all enterotoxins, most often detecting only those of the classical enterotoxin group, as in the case of the reverse passive latex agglutination (RPLA) method [2].

Staphylococci are a highly prevalent group of microorganisms both on farms and in humans, animals (cows), and their interaction environment. They enter the raw material directly from the cow’s udder, milking equipment, or production workers, among others. They are considered the main cause of mastitis in cows, and therefore raw milk, as well as dairy products made from raw milk, can be a source of staphylococci and toxic products of their metabolism, and therefore have a significant role associated with outbreaks of foodborne diseases in humans [18,19]. Due to the current trend in the consumption of raw milk and dairy products made from raw milk, it is necessary to conduct research on the microbiological safety of these products [20]. Therefore, the aim of this study the genotypic and phenotypic analysis of the enterotoxicity potential of *Staphylococcus* ssp. isolates isolated from raw milk and raw milk cheeses.

## 2. Results

### 2.1. Isolates Identification

A total of 75 *Staphylococcus* spp. isolates obtained from raw cow’s milk and raw milk cheeses were used in this study. Among them, 19 isolates were identified as *S. aureus* and 56 as CoNS. 

Among the 49 *Staphylococcus* spp. isolates isolated from raw milk samples, 6 (12.2%) were classified as *S. aureus* and 43 (87.8%) as CoNS. The identification of CoNS allowed us to classify eleven (22.4%) as *S. haemolyticus*, seven (14.3%) as *S. simulans*, six (12.2%) as *S. epidermidis*, five (10.2%) as *S. chromogenes*, five (10.2%) *S. warneri*, three (6.1%) as *S. hominis*, two (4.1%) as *S. sciuri* and single isolates belonged to the species *S. capitis*, *S. saprophyticus*, *S. succinus* and *S. xylosus*.

Among the 26 isolates of *Staphylococcus* spp. isolated from cheese samples, 13 (50.0%) were classified as *S. aureus* and 13 (50.0%) as CoNS. The identification of CoNS allowed us to classify seven (26.9%) as *S. simulans*, five (19.2%) as *S. epidermidis* and one (3.8%) as *S. haemolyticus*. The results of the identification and source of isolation of each isolate are shown in Table S1.

### 2.2. Genotypic Characterization of Enterotoxigenic Potential of Staphylococcal Isolates

A total of 73 (97.3%) isolates showed the presence of at least one gene encoding the ability to produce enterotoxins. Only two isolates from raw milk (*S. epidermidis* and *S. hominis*) were negative for all tested genes.

Both *S. aureus* and CoNS contained genes from a group of classical enterotoxins, non-classical enterotoxins, exfoliative toxins, and toxic shock toxin. The presence of toxin genes differed between individual species. All isolates of *S. aureus*, *S. capitis*, *S. chromogenes*, *S. haemolyticus*, *S. saprophyticus*, *S. sciuri*, *S. simulans*, *S. succinus*, *S. warneri* and *S. xylosus* were positive for toxin genes.

**Figure 1:**
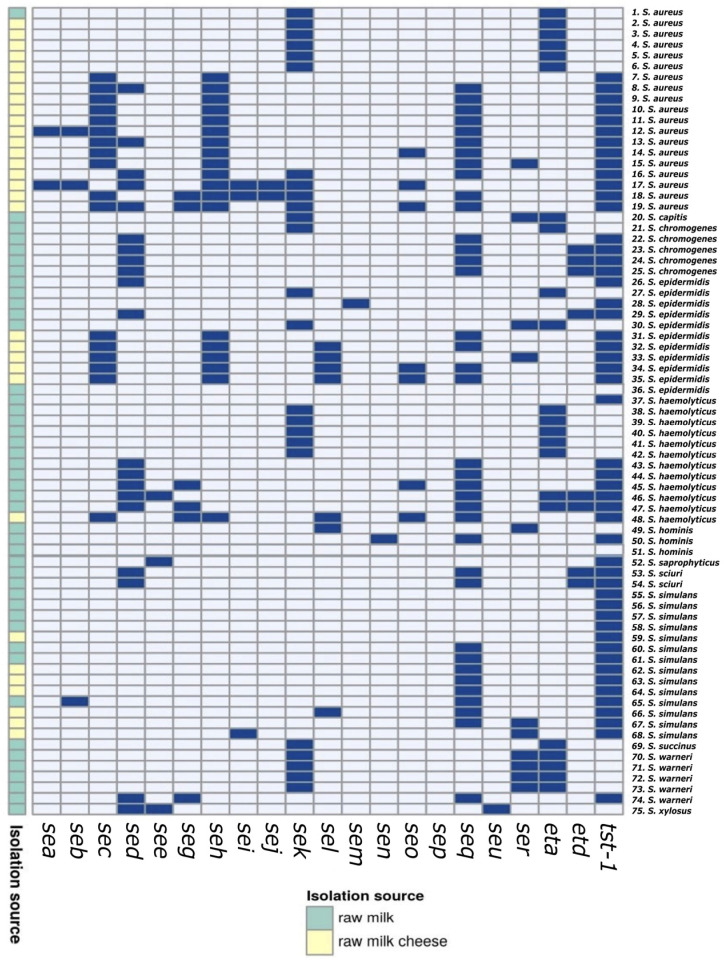
Presence of genetic determinants of enterotoxicity in staphylococcal isolates. (Abbreviation: dark blue—presence, light blue—absence). Results were visualized using the ClustVis visualizing tool (https://biit.cs.ut.ee/clustvis/, accessed on 18 August 2023).

Among isolates from raw milk samples, genes encoding classical SEs were detected—*seb* (1/2.1%), *sed* (15/31.9%) and *see* (3/6.4%). The most frequent genes were: *tst-1* (25/53.2%), *eta* (22/46.8%) and *sek* (20/42.6%). The following determinants of classical SEs were detected among isolates from cheese samples: *sea* (2/7.7%), *sec* (17/65.4%) and *sed* (5/19.2%). The most frequent genes in these isolates were: *seq* (20/76.9%) and *seh* (19/73.1%). The *tst-1* gene (26/34.7%) encoding toxic shock toxin was found in each of the isolates analyzed from the cheese samples. Among the isolates isolated from cheese, no genes encoding exfoliative toxins were found. The *sep* gene was not identified in any of the isolates tested (Figure 1).

A total of thirty-six different enterotoxicity profiles were observed (Table 1). The presence of up to eight different genes encoding classical and non-classical SEs, as well as exfoliative toxins and toxic shock toxin, were determined in the staphylococcal genome. The largest number of isolates contained a combination of two enterotoxin genes (23/30.7%). The most frequent toxin gene combination was *seq*, *eta* was present in fourteen isolates (18.7%) and *seq*, *ser*, *eta* was present in six isolates (8.0%) (from raw milk samples). Six isolates (five isolated from raw milk samples and one from cheese) (8.0%) had only the *tst-1* gene. The *seq*, *tst-1* gene combination (5/6.7%) was observed in both isolates from raw milk samples (2/2.6%) and isolates from cheese samples (3/4.0%).

CoNS contained enterotoxicity genes that were not found in *S. aureus* isolates. They were *seb*, *see*, *sel*, *sem*, *sen*, and *etd*; on the other hand *sea*, *sej* genes were found only in the *S. aureus* isolates. For the CoNS group, the largest number of enterotoxigenic genes was identified in the genome of *S. epidermidis* isolates. Two genes encoding classical SEs (*seb*, *see*) and several other genes encoding enterotoxicity (*etd*, *eta*, *sem*, *sen*, *seu*, *sep*) were not detected in *S. aureus*.

### 2.3. Phenotypic Ability to Produce Enterotoxins—SET-RPLA Method

Isolates in which genes encoding classical SEs (*sea*, *seb*, *sec*, *sed*) were identified were subjected to phenotypic analysis of the ability to produce enterotoxins using the SET-RPLA kit. Of the thirty-six isolates (48.0%) containing genes encoding classical SEs, only one of the tested isolates (2.8%) had this ability confirmed. This was an isolate of *S. aureus* isolated from raw milk cheese sample, which had the *sea* gene (encoding SEA enterotoxin) and the *sed* gene (responsible for encoding SED enterotoxin). This isolate was found to have the ability to produce SED enterotoxin.

## 3. Discussion

Milk and dairy products, including cheeses made from raw milk, are a reservoir of potentially dangerous microorganisms carrying a variety of factors that can affect their pathogenicity. Staphylococci are one of the most prevalent groups of microorganisms on farms as well as in humans and animals and in the environment of their interaction [18]. Staphylococci are responsible for staphylococcal food poisoning (SFP) related to the consumption of milk and dairy products, among others [21]. Studies indicate that 95.0% of SFP is associated with genes encoding SEs [22], with the main role of enterotoxicity attributed to *S*. *aureus* [23]. The literature data points to the increasing prevalence of various virulence factors in bacteria, particularly CoNS. This includes the identification of genes responsible for the production of staphylococcal enterotoxins in CoNS [2,15,24,25,26,27,28,29,30]. In the study, the presence of one or more genes that encode the ability to produce enterotoxins in a total of 73 (97.3%) isolates was found, including all *S*. *aureus* isolates. Moreover, both *S*. *aureus* and CoNS isolates contained genes from the group of classical and non-classical SEs, exfoliative toxins and toxic shock toxin. The literature reports a lower frequency of genes encoding enterotoxins among isolates from raw milk and raw milk cheeses (21.0, 80.0%). Genes encoding classical SEs (*sea*, *seb*, *sec*, *sed*), non-classical SEs (*sej*, *seg*, *sei*, *ser*, *sem*, *sen*, *seo*, *seh*, *sej*, *sep*) and toxic shock toxin (*tst*-*1*) were identified in the isolated samples [6,15,26,30,31,32,33,34,35]. In the current study, 14 different genes (*seb*, *sed*, *see*, *sek*, *sem*, *sel*, *seo*, *sen*, *seg*, *seq*, *ser*, *etd*, *eta*, *tst*-*1*) were detected in strains isolated from raw milk samples, while 14 different genes (*sea*, *sec*, *sed*, *sek*, *seh*, *sel*, *seo*, *seg*, *seq*, *sej*, *sei*, *ser*, *tst*-*1*) were detected in strains from raw milk cheeses. Several genes were not detected in the genomes of staphylococci from raw milk samples—two encoding classical enterotoxins (*sea*, *sec*) and five encoding non-classical enterotoxins (*seh*, *sej*, *sei*, *seu*, *sep*). In the case of staphylococci from raw milk cheese samples, the genes encoding exfoliative toxins (*eta*, *etd*) and two classical enterotoxins (*seb*, *see*), and several non-classical enterotoxins (*sem*, *sen*, *seu*, *sep*) were not identified. It should be noted that non-classical enterotoxins were significantly more frequently identified in the genome of CoNS rats than classical enterotoxins, regardless of the site of staphylococcal isolation. The variation in the prevalence of staphylococci among the various groups and, consequently, the presence of genetic determinants of enterotoxicity is due to several factors, including the use of different breeding and production practices in milk processing plants, the use of different hygiene measures, geographic location, or different microbial isolation techniques [36,37,38,39,40].

The present study’s results show the occurrence of staphylococcal isolates having the same combination of SAg. A total of seven different combinations of profiles of the same enterotoxin-encoding genes in more than one isolate were identified. Research conducted by Fijałkowski et al. (2014) [6] also showed the presence of *S*. *aureus* (isolated from cow’s milk) containing the identical genes combination of the SAg (*seg*, *sei*, *sem*, *sen*, *seo*). Researchers report that the same set of genes is characteristic of one clonal type of bacteria, which may influence the faster spread of specific staphylococcal genotypes, characterized by specific virulence and resistance to the defense mechanisms of the microorganism’s host [6,41,42,43]. The current research also showed that genes tagged in *S*. *aureus* were also detected in CoNS, and the whole number of various genes identified in CoNS was seven times higher than in *S*. *aureus*. This suggests the possibility of SAg gene transfer between staphylococci of different groups and species. The presence of SAg genes in CoNS confirms the possibility of their involvement in causing food poisoning, identified until recently only with the activity of *S*. *aureus* [6]. It should be noted that the results of the current study shows that isolated strains of the genome have a higher percentage of genes that encode non-classical enterotoxins. These enterotoxins, similar to classical SEs, pose a significant threat to public health. Therefore, controlling their presence in food isolates is necessary [44,45,46,47].

Some of the most important genetic determinants affecting the enterotoxicity potential of staphylococcal isolates are genes encoding the production of toxic shock toxin (*tst*-*1*) and exfoliative toxins (*eta*-*etd*). These toxins have the potential to cause a variety of symptoms in both humans and animals, as well as fever, rash, vascular disorders, toxic shock syndrome, multi-organ dysfunction and decreased blood pressure [48,49]. In the current study, the presence of the *tst*-*1* gene was found in 26 (51.0%) isolates from raw milk and in all isolates (*n* = 26) from cheeses made from raw milk. In contrast, genes encoding exfoliative toxins were present only in isolates from raw milk—22 (46.8%) and 8 (17.0%) for the *eta* and *etd* genes, respectively. The *tst*-*1* gene is responsible for encoding the toxic shock toxin, which *S*. *aureus* uses to colonize more easily [50]. The *tst*-*1* gene is more prevalent in strains from the animal community (including cows) than those from humans [35,51], which was confirmed in the current study.

Testing for the presence of genetic determinants of classical and non-classical SE and SE-like among strains from food is not routinely conducted at present, so it is currently difficult to accurately assess the risks they may have on public health. Continued research is needed to better understand these risks and assess their impact on the incidence of foodborne diseases [23,33,38].

Among SEs, the classical SEs is the most common cause of SFP worldwide [52]. Isolates showing the presence of genes encoding classical enterotoxins in their genome were tested for toxin production using a commercial SET-RPLA kit. In the current study, out of thirty-six isolates (48.0%) containing genes encoding classical toxins (*sea*, *seb*, *sec*, *sed*), only one isolate (2.8%) was found capable of producing SED toxin, associated with the presence of the *sed* gene in the genome of the isolate. The literature data report that the number of PCR-positive isolates (having genes encoding SEs) is noticeably higher than the number of isolates found to detect enterotoxins using the SET-RPLA method [53,54]. Discrepancies between methods may be due to the point mutation of genes, resulting in the transformation of enterotoxigenic genes into quiescent genes with expression defects; however, genes first silenced could be silenced temporarily—under environmental stress, gene expression could change [54,55,56]. In contrast, differences may also result from insufficient production of enterotoxin by isolates or production of toxin below the detection limit of the RPLA method. The SET-RPLA kit is considered a semi-quantitative assay that, according to the manufacturer’s data and previous studies [57], has a limit of detection (LOD) of 0.5 ng/mL of enterotoxin. The literature indicates a substitution in ongoing research from serological methods (including RPLA) to modern molecular biology methods (including real-time immunoquantitative PCR (iqPCR), chromatographic methods, immunoassays (including ELISA), chemiluminescence (CL) method, surface plasmon resonance (SPR) immunoassays (including sandwich SPR immunoassay), or aptamer-based bioassays (including FRET bioassay) [58,59]. These methods have much higher specificity and sensitivity for detecting enterotoxin than conventional immunosensor-based methods of <10 pg/mL for SEB (using iqPCR) [60], 3 pmol/mL for SEB (using liquid chromatography electrospray ionization mass spectrometry (LC-ESI-MS)) [61] 0.05–1 ng/mL for SEA and SEB (using colorimetric capture ELISA) [62] 3.2 pg/mL for SEA (using microplate CL enzyme immunoassay) [63], 0.01 ng/mL for SEB (using gold nanoparticle-based CL immunosensor) [64], 100 pg/mL for SEB (using SPR immunosensor based on antibody-coated super paramagnetic nanobeads) [65], 0.3 pg/mL for SEB (using FRET bioassay using an aptamer-affinity method coupled with up conversion nanoparticles (UCNPs)) [66], depending on the matrix used (pure cultures or food samples).

## 4. Conclusions

Analysis of the genetic potential enterotoxin production among the tested staphylococcal isolates revealed a high percentage of *S*. *aureus* and CoNS possessing genes encoding the ability to produce enterotoxins from both raw milk and raw milk cheese samples. The staphylococcal isolates tested contained genes encoding both classical and non-classical enterotoxins, as well as toxic shock toxin and exfoliative toxins. However, while a high percentage of isolates included genes encoding classical enterotoxins, only a small percentage showed actual toxin production. The differences in the number of PCR-positive isolates (having SE-coding genes) and actual toxin production may be due to the limit of detection of the detection method used or PCR related shortcomings. This indicates the need to use methods with high specificity and sensitivity for detecting enterotoxin production in future studies.

## 5. Materials and Methods

### 5.1. Isolates Identification

For this study, seventy-five isolates of *Staphylococcus* spp. were tested. All isolates belong to the isolate culture collection of the Department of Food Microbiology, Meat Technology and Chemistry, University of Warmia and Mazury (Olsztyn, Poland), including forty-nine isolates from fifty-three samples of raw cow’s milk from healthy cows of the Holstein Friesian breed from the farms located in central Poland and twenty-six isolates from twenty-eight samples of raw milk cheeses purchased in stores and hypermarkets in the Olsztyn (Poland). Briefly, 10 mL (10 g) was transferred to a flask containing 90 mL of Giolitti–Cantoni broth (Merck, Darmstadt, Germany) and incubated (37 °C/24 h). After incubation, cultures were inoculated onto Baird-Parker agar medium (Merck, Darmstadt, Germany) for isolation of *Staphylococcus* spp. Then, 1–5 different colonies were taken and streaked on Rabbit Plasma Fibrinogen (RPF) agar (BioMérieux, Marcy-l’Étoile, France). The culture was designed to obtain differentiation of the obtained staphylococcus isolates in terms of coagulase production.

Before further analysis, the isolates were streaked on TSA plates (Tryptic Soy Agar; Merck, Darmstadt, Germany) and identified using MALDI-TOF MS (matrix-assisted laser desorption and ionization-time of flight) according to manufacturer’s protocols, as described previously in our work [67,68,69].

### 5.2. Genotypic Characterization of Enterotoxigenic Potential of Staphylococcal Isolates

#### 5.2.1. DNA Extraction

The isolates of *Staphylococcus* ssp. had their genetic material extracted using Genomic Mini DNA Isolation Kit (A&A Biotechnology, Gdańsk, Poland) according to the manufacturer’s protocol, as in previous studies [70]. 

#### 5.2.2. Detection of Staphylococcal Superantigens by Multiplex PCR

Each isolate was tested for the presence of twenty-one genes encoding: classical and non-classical staphylococcal enterotoxins (*sea*, *seb*, *sec*, *sed*, *see*, *seg*, *seh*, *sei*, *sej*, *sek*, *sel*, *sem*, *sen*, *seo*, *sep*, *seq*, *seu*, *ser*), exfoliative toxins (*eta*, *etd*) and the *tst*-1 gene using multiplex PCR according to the methodology described previously [2,71,72,73,74]. The multiplex PCR for SAg genes was performed in a thermocycler (Mastercycler nexus GX2/GX2e; Eppendorf, Germany). The primer sequences and thermal process conditions are shown in Tables S2 and S3. A mixture of genomic DNA from seven *S. aureus* reference isolates was used as a positive control for each multiplex PCR reaction, while genomic DNA from *S. aureus* without SAg genes was used as a negative control (Table 2).

### 5.3. Phenotypic Ability to Produce (RPLA Method)

Enterotoxins produced by staphylococci in culture media were detected by the Reverse Passive Latex Agglutination (SET-RPLA) method. The SET-RPLA Toxin Detection Kit (Oxoid, Basingstoke, UK) uses antienterotoxins monoclonal antibodies and the sensitivity of detection for classical SEs (SEA-SED) is 0.5 ng/mL [57]. For the study, the selected isolates were those in which genotyping multiplex PCR identified genes encoding SE (*sea*, *seb*, *sec*, *sed*). Briefly, Brain Heart Infusion (BHI) broth (Merck, Darmstadt, Germany) was inoculated with one colony of each of the isolates analyzed and cultured at 37 °C for 24 h. A total of 25 μL of culture were then placed in 96-well V-bottom plates and the same amount of latex-sensitized particle solution was added and incubated (21 ± 1 °C/24 h). The latex particle agglutination observed on the bottom was reported as a positive result. The result was given as the presence/absence of SE production by the staphylococcal isolates.

## Figures and Tables

**Table 1:** The profiles of enterotoxins (classical and non-classical), exfoliative toxins (*eta-etd*) and toxic shock syndrome toxin-1 (*tst-1*) genes among the staphylococcal isolates.

No.	Toxin Genes Occurrence	Source of Isolate (Number of Isolates)	Species (Number of Isolates) (%)	Total (Number of Isolates) (%)	Number of Genes
1	*tst-1*	Raw milk (5), raw milk cheeses (1)	6 (8.0)	6 (8.0)	1
2	*sed*, *tst-1*	Raw milk (1)	1 (1.3)	23 (30.7)	2
3	*see*, *tst-1*	Raw milk (1)	1 (1.3)		
4	*sek*, *eta*	Raw milk (14)	14 (18.7)		
5	*sel*, *ser*	Raw milk (1)	1 (1.3)		
6	*sem*, *tst-1*	Raw milk (1)	1 (1.3)		
7	*seq*, *tst-1*	Raw milk (2), raw milk cheese (3)	5 (6.7)		
8	*seb*, *seq*, *tst-1*	Raw milk (1)	1 (1.3)	17 (22.7)	3
9	*sec*, *seh*, *tst-1*	Raw milk cheese (1)	1 (1.3)		
10	*sed*, *etd*, *tst-1*	Raw milk (1)	1 (1.3)		
11	*sed*, *see*, *seu*	Raw milk (1)	1 (1.3)		
12	*sed*, *seq*, *tst-1*	Raw milk (3)	3 (4.0)		
13	*sei*, *ser*, *tst-1*	Raw milk cheese (1)	1 (1.3)		
14	*sek*, *ser*, *eta*	Raw milk (6)	6 (8.0)		
15	*sel*, *seq*, *tst-1*	Raw milk cheese (1)	1 (1.3)		
16	*sen*, *seq*, *tst-1*	Raw milk (1)	1 (1.3)		
17	*seq*, *ser*, *tst-1*	Raw milk cheese (1)	1 (1.3)		
18	*sec*, *sed*, *seh*, *tst-1*	Raw milk cheese (1)	1 (1.3)	11 (14.7)	4
19	*sec*, *seh*, *seq*, *tst-1*	Raw milk cheese (4)	4 (5.4)		
20	*sed*, *seg*, *seq*, *tst-1*	Raw milk (1)	1 (1.3)		
21	*sed*, *seq*, *etd*, *tst-1*	Raw milk (5)	5 (6.7)		
22	*sea*, *sec*, *seh*, *seq*, *tst-1*	Raw milk cheese (1)	1 (1.3)	8 (10.7)	5
23	*sec*, *sed*, *seh*, *seq*, *tst-1*	Raw milk cheese (1)	1 (1.3)		
24	*sec*, *seh*, *sel*, *seq*, *tst-1*	Raw milk cheese (1)	1 (1.3)		
25	*sec*, *seh*, *sel*, *ser*, *tst-1*	Raw milk cheese (1)	1 (1.3)		
26	*sec*, *seh*, *seo*, *seq tst-1*	Raw milk cheese (1)	1 (1.3)		
27	*sec*, *seh*, *seq*, *ser*, *tst-1*	Raw milk cheese (1)	1 (1.3)		
28	*sed*, *seh*, *sek*, *seq*, *tst-1*	Raw milk cheese (1)	1 (1.3)		
29	*sed*, *seo*, *seg*, *seq*, *tst-1*	Raw milk cheese (1)	1 (1.3)		
30	*sec*, *seh*, *sel*, *seo*, *seq*, *tst-1*	Raw milk cheese (2)	2 (2.6)	4 (5.3)	6
31	*sed*, *see*, *seq*, *etd*, *eta*, *tst-1*	Raw milk (1)	1 (1.3)		
32	*sed*, *seg*, *seq*, *etd*, *eta*, *tst-1*	Raw milk (1)	1 (1.3)		
33	*sea*, *sed*, *seh*, *sei*, *sej*, *selo*, *tst-1*	Raw milk cheese (1)	1 (1.3)	2 (2.7)	7
34	*sec*, *seg*, *seh*, *sell*, *seo*, *seq*, *tst-1*	Raw milk cheese (1)	1 (1.3)		
35	*sec*, *sed*, *seg*, *seh*, *selk*, *seo*, *seq*, *tst-1*	Raw milk cheese (1)	1 (1.3)	2 (2.7)	8
36	*sec*, *seg*, *seh*, *sej*, *sek*, *seq*, *sei*, *tst-1*	Raw milk cheese (1)	1 (1.3)		
	Negative for toxin genes	Raw milk (2)	2 (2.6)	2 (2.7)	0
	Total		75 (100.0)	75 (100.0)	

**Table 2:** *S. aureus* control strains were used in this study.

Strains	Superantigen Gene (SAg)	References
FRI913	*sea*, *sec*, *see*, *sek*, *sel*, *seq*, *tst-1*	[75]
FRI137	*sec*, *seh*, *sel*, *seu*	
TY114	*etd*	
A920210	*eta*	
Col	*seb*, *sek*, *seq*	
FRI1151m	*sed*, *sej*, *ser*	[73]
N315	*sec*, *seg*, *sei*, *sel*, *sem*, *sen*, *seo*, *sep*, *tst1*	[76]
8325-4	No genes of SAg	[72]

## Data Availability

The data presented in this study are available in this article and Supplementary Materials.

## Supplementary Materials

The following supporting information can be downloaded at: https://www.mdpi.com/article/10.3390/toxins16010017/s1, Table S1. Identification results and source of isolates used in this study. Table S2. Primers and expected size of PCR products of investigated genes. Table S3. The thermal process conditions of multiplex PCR reaction of investigated genes.
