# Supplementary material for: Identification of the Enterotoxigenic Potential of Staphylococcus spp. from Raw Milk and Raw Milk Cheeses
Source: Toxins (Basel). 2023 Dec 28;16(1):17. doi: 10.3390/toxins16010017 (PMC10819113; doi:10.3390/toxins16010017)
Supplement: Supplementary file 1 [file toxins-16-00017-s001.zip › toxins-2754564-supplementary.pdf]

Identification of the Enterotoxigenic Potential of *Staphylococcus* spp. from Raw Milk and Raw Milk Cheeses

Patryk Wiśniewski, Joanna Gajewska, Anna Zadernowska and Wioleta Chajęcka-Wierzchowska

Table S1. Identification results and source of isolates used in this study.

No.	Identification number	MALDI	Source
1.	65G	*Staphylococcus aureus*	Raw milk – sample 1
2.	71G	*Staphylococcus aureus*	Raw milk – sample 2
3.	72G	*Staphylococcus aureus*	Raw milk – sample 3
4.	73G ż	*Staphylococcus aureus*	Raw milk – sample 4
5.	75G	*Staphylococcus aureus*	Raw milk – sample 5
6.	77G	*Staphylococcus aureus*	Raw milk – sample 6
7.	56G	*Staphylococcus capitis*	Raw milk – sample 7
8.	9	*Staphylococcus chromogenes*	Raw milk – sample 8
9.	10	*Staphylococcus chromogenes*	Raw milk – sample 8
10.	14	*Staphylococcus chromogenes*	Raw milk – sample 9
11.	79G	*Staphylococcus chromogenes*	Raw milk – sample 10
12.	70G	*Staphylococcus epidermidis*	Raw milk – sample 11
13.	74G	*Staphylococcus epidermidis*	Raw milk – sample 4
14.	80G	*Staphylococcus epidermidis*	Raw milk – sample 12
15.	11	*Staphylococcus epidermidis*	Raw milk – sample 13
16.	16	*Staphylococcus epidermidis*	Raw milk – sample 14
17.	17	*Staphylococcus epidermidis*	Raw milk – sample 15
18.	4	*Staphylococcus haemolyticus*	Raw milk – sample 16
19.	8	*Staphylococcus haemolyticus*	Raw milk – sample 17
20.	58G	*Staphylococcus haemolyticus*	Raw milk – sample 18
21.	62G	*Staphylococcus haemolyticus*	Raw milk – sample 19
22.	68G	*Staphylococcus haemolyticus*	Raw milk – sample 20
23.	6	*Staphylococcus haemolyticus*	Raw milk – sample 21
24.	12	*Staphylococcus haemolyticus*	Raw milk – sample 13
25.	63G	*Staphylococcus haemolyticus*	Raw milk – sample 22
26.	67G	*Staphylococcus haemolyticus*	Raw milk – sample 23
27.	33	*Staphylococcus hominis*	Raw milk – sample 24
28.	34	*Staphylococcus hominis*	Raw milk – sample 25
29.	35	*Staphylococcus hominis*	Raw milk – sample 26
30.	7	*Staphylococcus sciuri*	Raw milk – sample 27
31.	15	*Staphylococcus sciuri*	Raw milk – sample 21
32.	22	*Staphylococcus simulans*	Raw milk – sample 16
33.	73G b	*Staphylococcus succinus*	Raw milk – sample 4
34.	3	*Staphylococcus warneri*	Raw milk – sample 28
35.	78G	*Staphylococcus warneri*	Raw milk – sample 29
36.	57G	*Staphylococcus warneri*	Raw milk – sample 30
37.	61G	*Staphylococcus warneri*	Raw milk – sample 31
38.	76G	*Staphylococcus warneri*	Raw milk – sample 32
39.	1	*Staphylococcus xylosus*	Raw milk – sample 33
40.	13	*Staphylococcus chromogenes*	Raw milk – sample 13
41.	21	*Staphylococcus saprophyticus*	Raw milk – sample 16
42.	18	*Staphylococcus simulans*	Raw milk – sample 34
43.	19	*Staphylococcus simulans*	Raw milk – sample 28
44.	20	*Staphylococcus simulans*	Raw milk – sample 16
45.	23	*Staphylococcus simulans*	Raw milk – sample 16
46.	24	*Staphylococcus simulans*	Raw milk – sample 13
47.	25	*Staphylococcus simulans*	Raw milk – sample 13
48.	2	*Staphylococcus haemolyticus*	Raw milk – sample 34
49.	5	*Staphylococcus haemolyticus*	Raw milk – sample 35
50.	42G	*Staphylococcus epidermidis*	Parmigiano Reggiano
51.	44G	*Staphylococcus epidermidis*	Appenzeller cheese
52.	34G	*Staphylococcus aureus*	Brie Montasvy cheese
53.	35G	*Staphylococcus epidermidis*	Fourme D'ambert A.O.P. cheese
54.	16G	*Staphylococcus aureus*	Gruyere A.O.P. cheese
55.	15G	*Staphylococcus aureus*	Natural cheese
56.	8G	*Staphylococcus aureus*	Homemade natural cheese
57.	2G	*Staphylococcus aureus*	Homemade Spring cheese
58.	53G	*Staphylococcus aureus*	Cheeese with Chilli And Oregano
59.	17G	*Staphylococcus aureus*	Cheese with cumin
60.	7G	*Staphylococcus aureus*	Young Fudge Cheese
61.	18G	*Staphylococcus aureus*	Young Fudge Cheese With Tomato, Basil And Garlic cheese
62.	41G	*Staphylococcus aureus*	Cheese Rokblu Mild Cheese
63.	31G	*Staphylococcus aureus*	Fresh Cheese with Cumin
64.	54G	*Staphylococcus aureus*	Fresh Cheese with Cumin
65.	45G	*Staphylococcus aureus*	Homestead Cheese with Herbs Provençal
66.	43G	*Staphylococcus epidermidis*	Memel Blue cheese
67.	9G	*Staphylococcus epidermidis*	Ser Sant Mont Bergkäse
68.	40G	*Staphylococcus haemolyticus*	Ser Morbier Chnp
69.	28	*Staphylococcus simulans*	Royal Light cheese
70.	29	*Staphylococcus simulans*	Masdamer cheese

71.	26	*Staphylococcus simulans*	Wło cheese
72.	27	*Staphylococcus simulans*	Wlo cheese
73.	32	*Staphylococcus simulans*	Grillek cheese
74.	30	*Staphylococcus simulans*	Fresh Natural Cheese
75.	31	*Staphylococcus simulans*	Fresh Provençal Cheese

Table S2. Primers and expected size of PCR products of investigated genes.

	Gene	Primers	Sequences (5′ → 3′)	Produkt size (bp)	References
**Multiplex I**	*sea*	sea-1	GAA AAA AGT CTG AAT TGC AGG GAA CA	560	[63]
		sea-2	CAA ATA AAT CGT AAT TAA CCG AAG GTT C		
	seh	seh-1	CAA TCA CAT CAT ATG CGA AAG CAG	376	[63]
		she-2	CAT CTA CCC AAA CAT TAG CAC C		
	sec	sec-1	CTT GTA TGT ATG GAG GAA TAA CAA AAC ATG	275	[63]
		sec-2	CAT ATC ATA CCA AAA AGT ATT GCC GT		
	tst-1	tst-1	TTC ACT ATT TGT AAA AGT GTC AGA CCC ACT	180	[63]
		tst-2	TAC TAA TGA ATT TTT TTA TCG TAA GCC CTT		
**Multiplex II**	*sed*	sed-1	GAA TTA AGT AGT ACC GCG CTA AAT AAT ATG	492	[63]
		sed-2	GCT GTA TTT TTC CTC CGA GAG T		
	etd	etd-1	CAA ACT ATC ATG TAT CAA GGA TGG	358	[62]
		etd-2	CCA GAA TTT CCC GAC TCA G		
	eta	eta-1	ACT GTA GGA GCT AGT GCA TTT GT	190	[63]
		eta-2	TGG ATA CTT TTG TCT ATC TTT TTC ATC AAC		
	sek	sek-1	ATG CCA GCG CTC AAG GC	134	[64]
		sek-2	AGA TTC ATT TGA AAA TTG TAG TTG ATT AGC T		
		sek-3	TGC CAG CGC TCA AGG TG		
**Multiplex III**	*see*	see-1	CAA AGA AAT GCT TTA AGC AAT CTT AGG C	482	[63]
		see-2	CAC CTT ACC GCC AAA GCT G		
	seb	seb-1	ATT CTA TTA AGG ACA CTA AGT TAG GGA	404	[63]
		seb-2	ATC CCG TTT CAT AAG GCG AGT		
	sem	sem-1	CTA TTA ATC TTT GGG TTA ATG GAG AAC	326	[63]
		sem-2	TTC AGT TTC GAC AGT TTT GTT GTC AT		
	sel	sel-1	GCG ATG TAG GTC CAG GAA AC	234	[64]
		sel-2	CAT ATA TAG TAC GAG AGT TAG AAC CAT A		
	seo	seo-1	AGT TTG TGT AAG AAG TCA AGT GTA GA	180	[63]
		seo-2	ATC TTT AAA TTC AGC AGA TAT TCC ATC TAA C		
**Multiplex IV**	*sen*	sen-1	CGT GGC AAT TAG ACG AGT C	474	[64]
		sen-2	GAT TGA TYT TGA TGA TTA TKA G		
	seg	seg-1	TCT CCA CCT GTT GAA GG	323	[64]
		seg-2	AAG TGA TTG TCT ATT GTC G		
	seq	seq-1	ACC TGA AAA GCT TCA AGG A	204	[64]
		seq-2	CGC CAA CGT AAT TCC AC		
	sej	sej-1	TCA GAA CTG TTG TTC CGC TAG	138	[64]
		sej-2	GAA TTT TAC CAY CAA AGG TAC		
**Multiplex V**	*sei*	sei-1	CTY GAA TTT TCA ACM GGT AC	461	[64]
		sei-2	AGG CAG TCC ATC TCC TG		
	ser	ser-1	AGC GGT AAT AGC AGA AAA TG	363	[64]
		ser-2	TCT TGT ACC GTA ACC GTT TT		
	seu	seu-1	AAT GGC TCT AAA ATT GAT GG	215	[64]
		seu-2	ATT TGA TTT CCA TCA TGC TC		
	sep	sep-1	GAA TTG CAG GGA ACT GCT	182	[64]
		sep-2	GGC GGT GTC TTT TGA AC		

Table S3. The thermal process conditions of multiplex PCR reaction of investigated genes.

Step	PCR Phase	Temperature (°C)	Time (s)	Cycle
**1**	Initial denaturation	95	600	1
**2**	Denaturation	95	30	35
**3**	Annealing	55	45	
**4**	Extension	72	60	
**5**	Final extension	72	600	1
